# Cold In-Place Recycling Asphalt Mixtures: Laboratory Performance and Preliminary M-E Design Analysis

**DOI:** 10.3390/ma14082036

**Published:** 2021-04-18

**Authors:** Dongzhao Jin, Dongdong Ge, Siyu Chen, Tiankai Che, Hongfu Liu, Lance Malburg, Zhanping You

**Affiliations:** 1 Department of Civil and Environmental Engineering, Michigan Technological University, 1400 Townsend Drive, Houghton, MI 49931-1295, USA; dongj@mtu.edu (D.J.); dge1@mtu.edu (D.G.); siychen@mtu.edu (S.C.); tche@mtu.edu (T.C.); hliu16@mtu.edu (H.L.); 2 School of Transportation, Southeast University, Nanjing 211189, China; 3 School of Transportation and Logistics, Dalian University of Technology, Dalian 116024, China; 4 Dickinson County Road Commission, Iron Mountain, MI 49801, USA; lance@dickinsoncrc.com

**Keywords:** cold in-place recycling (CIR), disk-shaped compact tension (DCT), moisture-induced stress tester (MIST), dynamic modulus, dynamic shear rheometer (DSR), asphalt binder cracking device (ABCD)

## Abstract

Cold in-place recycling (CIR) asphalt mixtures are an attractive eco-friendly method for rehabilitating asphalt pavement. However, the on-site CIR asphalt mixture generally has a high air void because of the moisture content during construction, and the moisture susceptibility is vital for estimating the road service life. Therefore, the main purpose of this research is to characterize the effect of moisture on the high-temperature and low-temperature performance of a CIR asphalt mixture to predict CIR pavement distress based on a mechanistic–empirical (M-E) pavement design. Moisture conditioning was simulated by the moisture-induced stress tester (MIST). The moisture susceptibility performance of the CIR asphalt mixture (pre-mist and post-mist) was estimated by a dynamic modulus test and a disk-shaped compact tension (DCT) test. In addition, the standard solvent extraction test was used to obtain the reclaimed asphalt pavement (RAP) and CIR asphalt. Asphalt binder performance, including higher temperature and medium temperature performance, was evaluated by dynamic shear rheometer (DSR) equipment and low-temperature properties were estimated by the asphalt binder cracking device (ABCD). Then the predicted pavement distresses were estimated based on the pavement M-E design method. The experimental results revealed that (1) DCT and dynamic modulus tests are sensitive to moisture conditioning. The dynamic modulus decreased by 13% to 43% at various temperatures and frequencies, and the low-temperature cracking energy decreased by 20%. (2) RAP asphalt incorporated with asphalt emulsion decreased the high-temperature rutting resistance but improved the low-temperature anti-cracking and the fatigue life. The M-E design results showed that the RAP incorporated with asphalt emulsion reduced the international roughness index (IRI) and AC bottom-up fatigue predictions, while increasing the total rutting and AC rutting predictions. The moisture damage in the CIR pavement layer also did not significantly affect the predicted distress with low traffic volume. In summary, the implementation of CIR technology in the project improved low-temperature cracking and fatigue performance in the asphalt pavement. Meanwhile, the moisture damage of the CIR asphalt mixture accelerated high-temperature rutting and low-temperature cracking, but it may be acceptable when used for low-volume roads.

## 1. Introduction

Asphalt recycling has increased dramatically in the past several years [1,2]. The rehabilitation of pavement also has lots of techniques, and one eco-friendly methodology is to use a CIR asphalt mixture [3,4]. CIR is a procedure whereby the overlayer of asphalt pavement is milled and mixed with stabilizers like emulsified asphalt [5]. Early applications of CIR focused on low-volume pavements [6]. Cost-effectiveness was the primary factor for using CIR. Bradbury et al. [7] stated it was about one-third cheaper than the original hot asphalt pavement road in Ontario. Scholz et al. [8] paved a 50 mm thick asphalt concrete surface with CIR and saved about 40% of the total cost. Many researchers have focused on the compaction and mixing procedure of CIR mixtures especially relating to optimum moisture content. Anderson et al. [9] discovered that in order to obtain good road performance, especially regarding strength and anti-cracking properties, optimum moisture content was the key factor that affected the on-site compaction degree and even mixing. In general, the Marshall method is always chosen to obtain the optimum moisture content [10], but the relationship between moisture and density could also help obtain it [11]. Curing time was also a vital factor affecting the CIR asphalt mixture, which is typically cured in a 60 °C oven [12]. Woods et al. [13] implied that the criteria content of the moisture is 1.5% based on the moisture sensors at different depths in on-site CIR overlays.

Quantitative researchers have concentrated on the performance characterization test of the CIR asphalt mixture. Wu et al. [14] conducted asphalt concrete APA fatigue tests by using a more traditional theoretical analysis. Marshall stability has traditionally been the main criterion used to select design binder content for bituminous-stabilized CIR mixtures. Yan et al. [15] stated that APA test was conducted by many researchers to estimate anti-rutting performance, and he suggested that Marshall stability should be larger than at 6 kN at 40 °C. Du and Cross [16] used three types of CIR mixture (1.5% asphalt emulsion, 1.5% asphalt emulsion with hydrated lime, 1.5% asphalt emulsion with quick lime) to test rutting performance using APA tests, and the rut depth ranged from 0.37 to 0.67 cm. Indirect Tensile Strength has been used by many people to study CIR asphalt mixture performance. Yan et al. [15] suggested that the peak load at 15 °C should be larger than 0.5 MPa. Kavussi and Modarres [17] conducted an IDT test of CIR mixtures and figured out that the coefficient of variation is no larger than 0.1 in most cases; Thomas et al. [18] tested two types of CIR mixture by an IDT test and by creep compliance and strength to characterize low temperature cracking performance. Based on the above literature review, the performance was one of the vital factors in CIR asphalt mixtures. In this study, all loose materials used were obtained from Dickinson County Road 581, located on the Upper Peninsula of Michigan, where the lowest temperature was below 0 °C for almost half a year, indicating that low-temperature performance is important for road service. Meanwhile, CIR performance at high temperatures is important in summertime [19,20]. A CIR asphalt mixture commonly has higher air voids, which may cause moisture damage. Therefore, it is necessary to investigate the CIR asphalt mixture moisture resistance. 

The main purpose of this research is to characterize the effect of moisture on the high-temperature and low-temperature performance of CIR asphalt mixture and to predict pavement distress and deterioration based on a mechanistic–empirical pavement design. The low-temperature cracking performance of the CIR asphalt mixture was estimated by the DCT test. The dynamic modulus was conducted to evaluate the stress and strain response of the CIR asphalt mixture at various temperatures and frequencies. In addition, the Moisture-Induced Stress Tester (MIST) was used to simulate the pore pressure generated in a wet pavement under moving traffic loading. The standard solvent extraction test was used to obtain the reclaimed asphalt pavement (RAP) and CIR asphalt. The high-temperature and fatigue performances were characterized by a dynamic shear rheometer (DSR) equipment, while the low-temperature properties were investigated by the asphalt binder cracking device (ABCD). Then the predicted pavement distresses were estimated based on the M-E inputs.

## 2. Materials and Methods

### 2.1. Materials and Mixture Design

**Figure 1:**
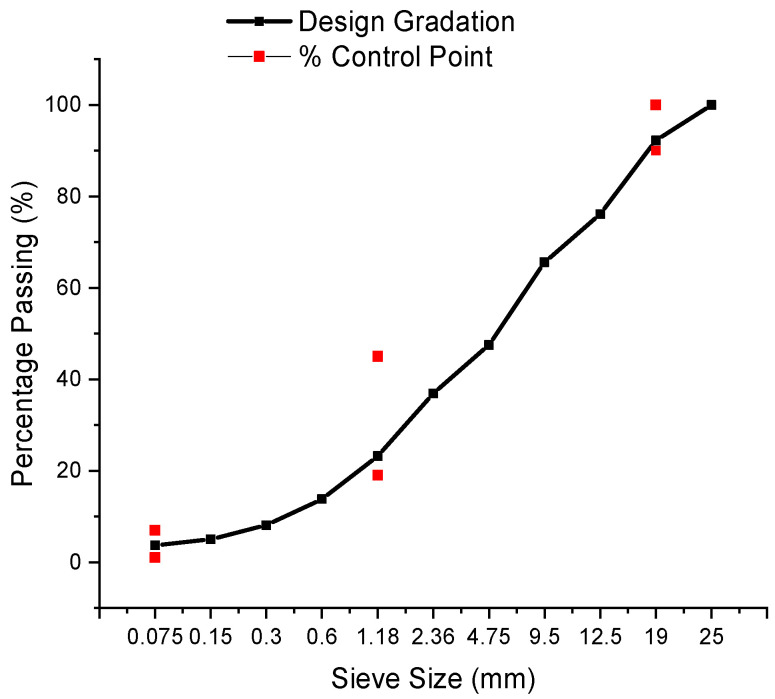
The gradation used in this study.

All the loose mixtures are obtained from Dickinson County Road 581 in Michigan, USA. The mix design was conducted by the Superpave mix design. The RAP materials obtained from the milled surface asphalt pavement layer were utilized with 2.5% emulsion content (64.8% residue content) and an additional 2% water content based on the mix design results shown in Table 1. The average bulk specific gravity is 2.129. The gradation used in this study is shown in Figure 1. The Superpave gyratory compactor (SGC, manufacturer: PINE test equipment, Grove City, PA, USA) gyration time is 30 for CIR mixture in nearly all US states [21], so the gyration time was 30 and the mass was cured at 60 °C for 48 h until it was constant. The average air voids were 13%. The SGC compaction procedure was followed the AASHTO PP 60-09 specification [22].

### 2.2. Experimental Program

**Figure 2:**
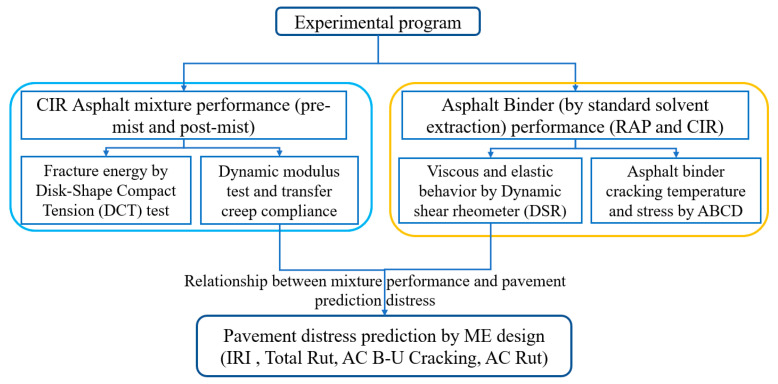
The technical flowchart of this study.

This section concerns the laboratory mixture experiments, which included the moisture conditioning test (MIST), the dynamic modulus test, and the low-temperature DCT test. The laboratory binder experiments, including the standard solvent extraction test, were used to obtain the reclaimed asphalt pavement (RAP) and CIR binder. The dynamic shear rheometer (DSR, manufacturer: Anton Paar, North Ryde, Australia), the asphalt binder cracking device (ABCD) test, and then the prediction pavement distress were estimated by the M-E design. The technical flowchart is shown in Figure 2.

### 2.3. Asphalt Mixture Test Program

#### 2.3.1. Moisture Conditioning via MIST

A CIR asphalt mixture generally shows a high air void, and it causes the asphalt mixture to be more sensitive to moisture damage [23]. Therefore, there is a need to investigate the moisture conditioning performance of the CIR asphalt mixture. The Moisture Induced Stress Tester (MIST, manufacturer: InstroTek, Grand Rapids, MI, USA) is designed to be a quick and logical method for testing the moisture damage susceptibility of asphalt mixture, which is specified in ASTM D7870 [24]. The samples for the dynamic modulus and disk-shaped compact tension tests were conditioned by MIST. It should be noted that the moisture conditioning was set for 500 cycles at 40 °C for the CIR asphalt mixture. Specifically, the CIR asphalt mixture without moisture conditioning was labeled as “pre-mist”, while the CIR asphalt mixture under moisture conditioning was labeled as “post-mist”.

#### 2.3.2. Low-Temperature Cracking Test

**Figure 3:**
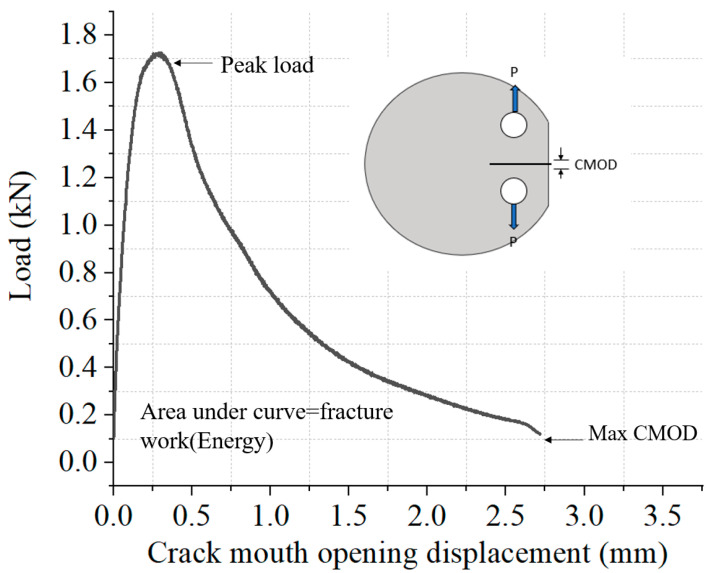
Demonstration of the DCT test procedure.

Dickinson County Road 581 is located on the Upper Peninsula of Michigan where the lowest temperature was less than 0 °C for almost half a year, which means that low-temperature performance is vital for road service. The DCT test is used to evaluate the low-temperature cracking resistance of the asphalt mixture and is based on ASTM D7313 [25], “Standard Test Method for Determining Fracture Energy of Asphalt Mixtures Using the Disk-Shaped Compact Tension Geometry”. The test is generally used to obtain the fracture energy, peak load, and maximum crack mouth-opening displacement (CMOD). The DCT test temperature for the CIR asphalt mixture was −12 °C based on the PG level. The test was run with a constant CMOD control mode, and the CMOD rate was 1 mm/min. The CMOD after the peak load reflected the propagation of cracking inside the sample during the DCT test. The specimen’s geometry for a DCT test is a cylinder with a diameter of 150 mm and height of 45 mm. The details of the DCT test are shown in Figure 3. It should be mentioned that at least three parallel samples were used for each test.

#### 2.3.3. Dynamic Modulus Test

**Figure 4:**
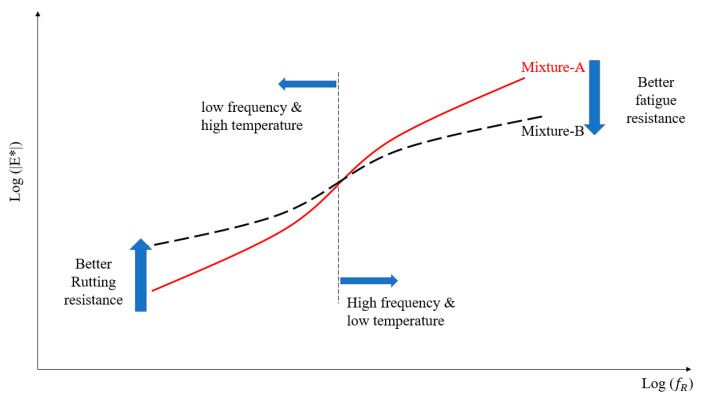
Demonstration of the dynamic modulus master curve.

The dynamic modulus could be used directly to reflect the stress and strain response by specific load [26,27]. The dynamic modulus (E*) test is conducted at temperatures of −10 °C, 10 °C, 21 °C, and 37 °C, and at loading frequencies of 0.1 Hz, 0.5 Hz, 1 Hz, 5 Hz, 10 Hz, and 25 Hz at each temperature. The UTM-100 equipment was used for this test, and it is specified in AASHTO T342. The temperature chamber of the UTM-100 machine can control the temperature from -15 to 60 °C. The diameter and height of the test specimens were 100 mm and 150 mm, respectively. The dynamic modulus (|E*|) reflected the elastic performance of the asphalt mixture, and the phase angle (δ) expressed the gap between the stress and the strain. The rutting parameter (|E*|/sin δ) and fatigue parameter (|E*|∙sin δ) could reflect the rutting and fatigue properties of the two types of asphalt mixtures. The master curve of the dynamic modulus was plotted for both the dynamic modulus of asphalt mixture and asphalt binder. It could be used to reveal the high-temperature performance and low-temperature performance. The details of the dynamic modulus master curve are shown in Figure 4. The specimen’s geometry for the dynamic modulus test is a cylinder with a diameter of 100 mm and height of 150 mm. It should be mentioned that at least three parallel samples were used for each test.

#### 2.3.4. Creep Compliance

Creep compliance at low temperature could be used to reflect the thermal cracking at the low temperature of the asphalt mixture. It is based on AASHTO T322 (2011) [28] “Determining the Creep Compliance and Strength of Hot Mix Asphalt (HMA) Using the Indirect Tensile Test Device”. Creep compliance would be estimated from the |E*| master curve using the procedure developed in 1999 by S.W. Park and R.A. Shapery [29]. The master curve of dynamic modulus |E*| could be used to characterize the linear viscoelastic behavior of asphalt mixtures and conversion between the time and frequency domains.

### 2.4. Asphalt Binder Test Program

#### 2.4.1. Standard Solvent Extraction 

The properties of CIR asphalt also show significant variants compared with RAP asphalt from the milled pavement. Therefore, there is a need to characterize the extraction of asphalt binder properties. The automatic asphalt analyzer (manufacturer: CONTROLS Inc., Chicago, IL, USA) was used for the washing of the CIR loose mixture (around 3.5 kg) with trichloroethylene (TCE) solvent, ultrasonic motion, simultaneous heating, and rotation of the drum lined with screening mesh, which is specified in ASTM D8159-19 [30]. The solvent mix incorporated with asphalt binder was moved to the rotary evaporator device to remove the solvents from the asphalt binder by evaporation extracts. Asphalt binder washed out from the mixture with the Automatic Asphalt Analyzer could be separated from the asphalt. In short, the extracted asphalt binder from the RAP loose mixture could be written as RAP, and the extracted asphalt from the CIR loose mixture could be written as CIR.

#### 2.4.2. Dynamic Shear Rheometer (DSR) 

The viscous and elastic property of asphalt at medium and high temperatures was characterized by dynamic shear rheometer equipment, which characterized high-temperature and fatigue performance. The high-temperature rheological properties were be conducted at a temperature of 34, 40, 46, 52, 58, 64, 70, 76, and 82 °C at loading frequencies of 0.628, 6.28, 9.99, 18.8, 31.4, and 62.8 rad/s for each temperature. The intermediate temperature was conducted at a temperature of 13, 16, 19, 22, and 25 °C at loading frequencies of 0.628, 6.28, 9.99, 18.8, 31.4, 62.8 rad/s for each temperature. The test procedure was based on the AASHTO 315 specification. At least three parallel samples were used for each test.

#### 2.4.3. Asphalt Binder Cracking Device (ABCD)

**Figure 5:**
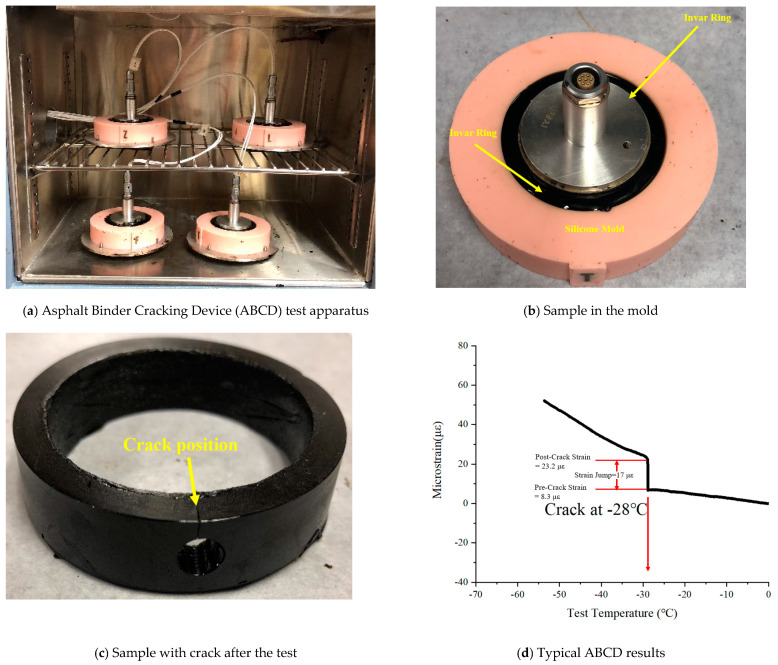
The Asphalt Binder Cracking Device (ABCD) test apparatus and procedure: (**a**) Asphalt Binder Cracking Device (ABCD) test apparatus; (**b**) Sample in the mold; (**c**) Sample with crack after the test; (**d**) Typical ABCD results.

The ABCD is a new empirical test for evaluating the low-temperature cracking potential of asphalt binder. The cracking temperature and cracking stress can be obtained from the test, which is specified in AASHTO TP 92(2014) [31]. Strain and temperature are recorded until the cracking starts. The ABCD system consists of an air-cooled environmental chamber that can automatically cool asphalt specimens at a constant rate (20 °C /h) from 25 °C to −60 °C. The ABCD invar rings are equipped with an electrical-strain reading gauge, temperature control sensor, and silicone rubber specimen molds. Typical ABCD setup and test results are shown in Figure 5. At least three parallel samples were used for each test.

### 2.5. Pavement Distress Prediction by Pavemeεnt M-E Design Analysis

**Figure 6:**
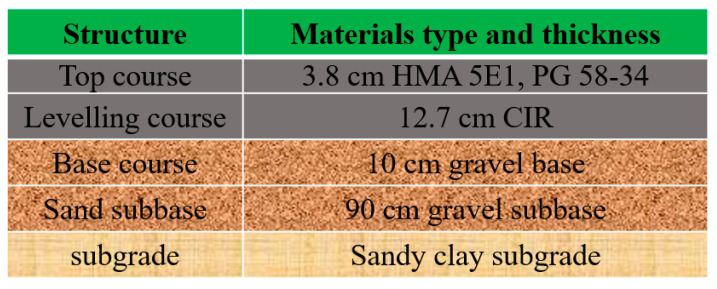
Demonstration of the structure and materials used in this study.

The M-E Design [29] was used to evaluate the difference in the asphalt binder performance between the RAP and CIR asphalt, especially for the permanent deformation and fatigue cracking. The dynamic modulus pre-mist and post-mist conditions were also used to predict the effect of moisture damage in the CIR asphalt layer on pavement distress. The test parameters were the following: vehicle growth rate was 0.5%, the design life was 20 years, design speed was 90 km/h, the annual average daily traffic (AADT) in 2019 was 778, design function of the traffic volume was compound, and the number of lanes was 2. The traffic value, including single axle, tandem axle, tridem axle, quad axle distribution, was set according to the Buch research report [30]. The climate was the near place according to the M-E used guide recommendation. The specific calibration factor was according to the Michigan DOT User Guide for Mechanistic–empirical Pavement Design 2020, and the pavement structure and thickness used in this road is shown in Figure 6.

## 3. Results and Discussions

### 3.1. Dynamic Modulus Test Results

**Figure 7:**
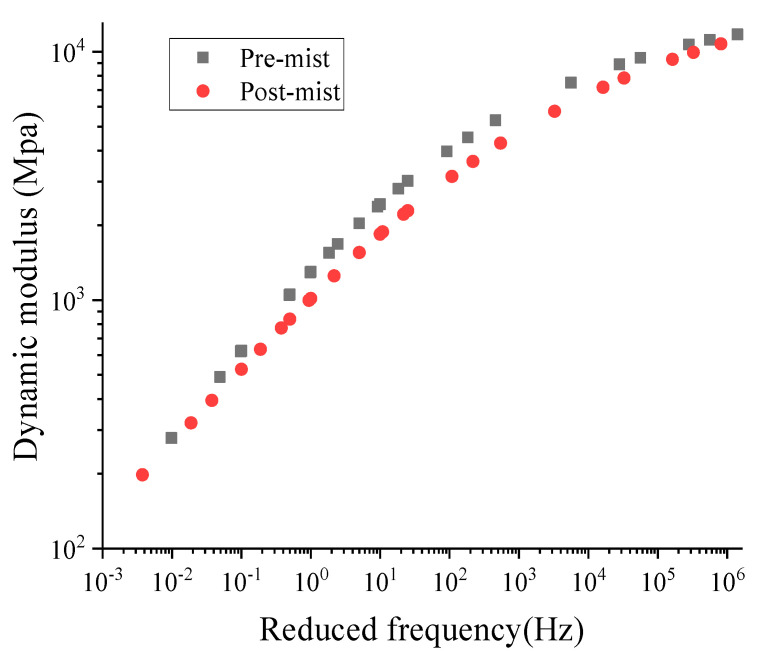
Master curve of dynamic modulus at 21 °C of pre-mist and post-mist CIR asphalt mixture.

Figure 7 shows the dynamic modulus master curve at a reference temperature of 21 °C for the pre-mist and post-mist CIR asphalt mixture. It was determined that the stiffness of all mixtures would decrease after moisture conditioning. As we all know, a lower reduced frequency represents slow traffic speeds or high pavement temperature, and a higher reduced frequency indicates high traffic speeds or low pavement temperature. Both the lower and high reduced frequencies showed that a reduction in stiffness in post-mist CIR asphalt mixture compared with the pre-mist CIR asphalt mixture.

**Figure 8:**
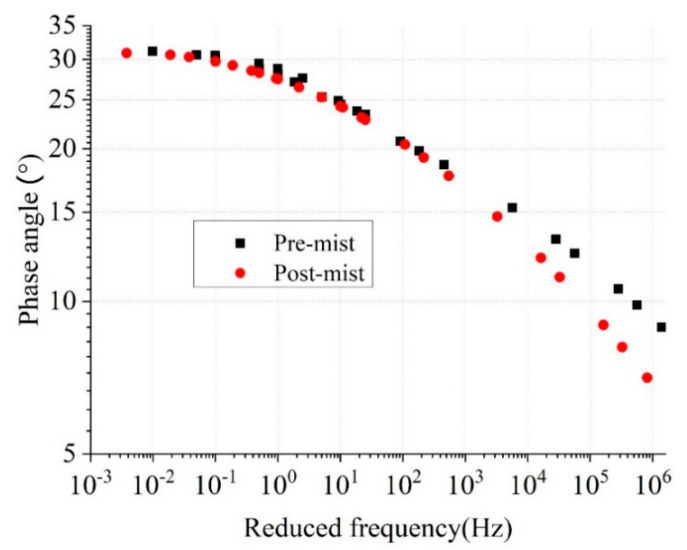
Master curve of phase angle at 21 °C of pre-mist and post-mist CIR asphalt mixture.

The phase-angle master curve at the reference temperature of 21 °C between the pre-mist and post-mist CIR is shown in Figure 8. It was apparent that the phase angle increased after MIST conditioning. At lower frequencies, the phase angle increased as little as 0.2 degrees, while it could be 1 degree at high frequencies. The reason is that after MIST conditioning, the asphalt mixture displayed more viscous action under loading. The results from the dynamic modulus test indicated that after the moisture damage, the asphalt mixture experienced a decrease in modulus and an improvement in phase angle, which meant that the asphalt mixture was softer after the moisture damage.

**Figure 9:**
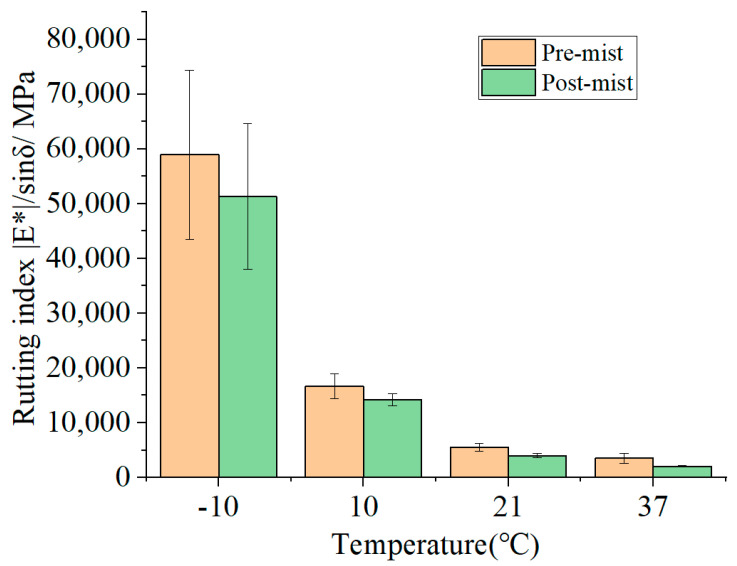
Rutting index at 10 Hz of pre-mist and post-mist CIR asphalt mixture.

The rutting index of the pre-mist and post-mist CIR mixtures at the loading frequency of 10Hz is displayed in Figure 9. The rutting parameter (|E*|/sinδ) quantifies the rutting property of the asphalt mixture. The MIST process reduced the rutting parameter of the mixture. For example, the rutting parameter of the asphalt mixture decreased by 13% at −10 °C and by 41% at 37 °C. This reduction effect was significant at high temperatures, which was very important because at high temperatures rutting was easier to accumulate on the pavement. The stiffness of the asphalt mixture decreased after fiber modification, thus weakening the rutting resistance of the asphalt mixture. It should be mentioned that the high value of error bar at −10 °C may be caused by moisture frozen in the mixture.

**Figure 10:**
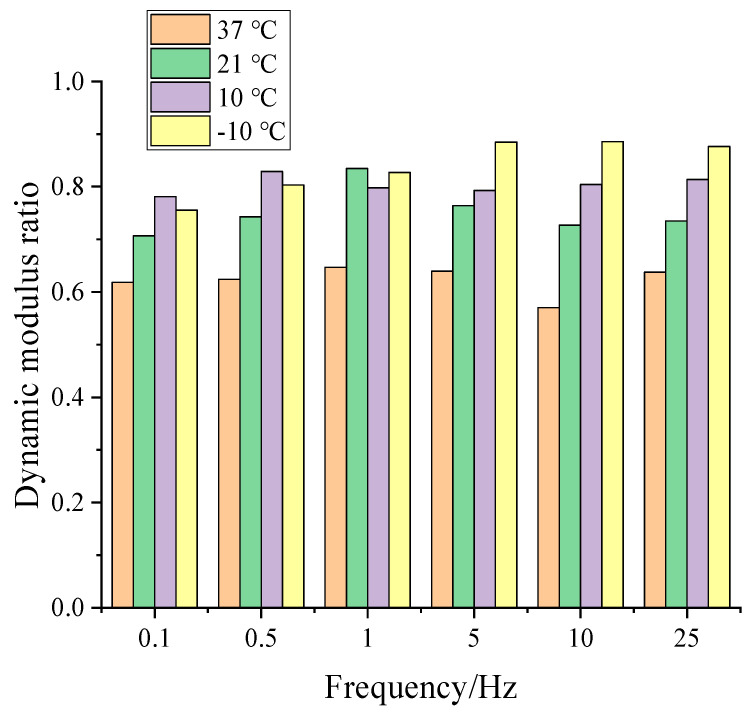
Dynamic modulus ratio of pre-mist and post-mist CIR mixture at 10Hz.

The moisture susceptibility of the pre-mist and post-mist CIR mixture was evaluated by the dynamic modulus ratio. For CIR, this was defined by the ratio of the dynamic modulus of pre-mist CIR mixture to that of the post-mist CIR mixture. The results of the various mixtures at different temperatures and frequencies are presented in Figure 10. The lower dynamic modulus ratio values were observed with a decrease in loading frequency and increase in temperature. The reduction in stiffness was more pronounced at lower frequencies, and after conditioning the material experienced a decrease in stiffness at all temperatures. These results indicated that asphalt mixtures were more affected by humidity regulation at lower traffic speeds and higher temperatures.

### 3.2. Creep Compliance

**Figure 11:**
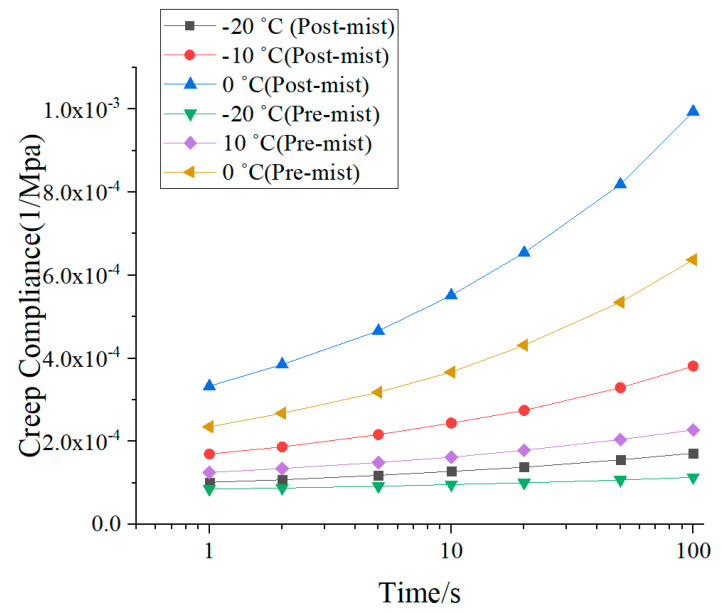
Creep compliance results of pre-mist and post-mist CIR asphalt mixture.

Creep compliance at low temperature was used to determine the cracking resistance for the asphalt mixture [32,33]. Creep compliance revealed low-temperature behavior and predicted the thermally induced cracking in asphalt pavement at low temperature in the U.S. [34]. The results for the pre-mist and post-mist CIR mixtures, are shown in Figure 11. The temperature increases caused the creep compliance to increase as did the duration time increase. It is clear that the post-mist CIR showed higher creep compliance under various temperatures and times. For example, it increased by 55% from 4.396 × 10^−6^ to 6.85 × 10^−6^. This meant that the stiffness of the post-mist CIR mixture decreased and the low temperature cracking resistance decreased compared with the pre-mist CIR mixture.

### 3.3. DCT Test Results

**Figure 12:**
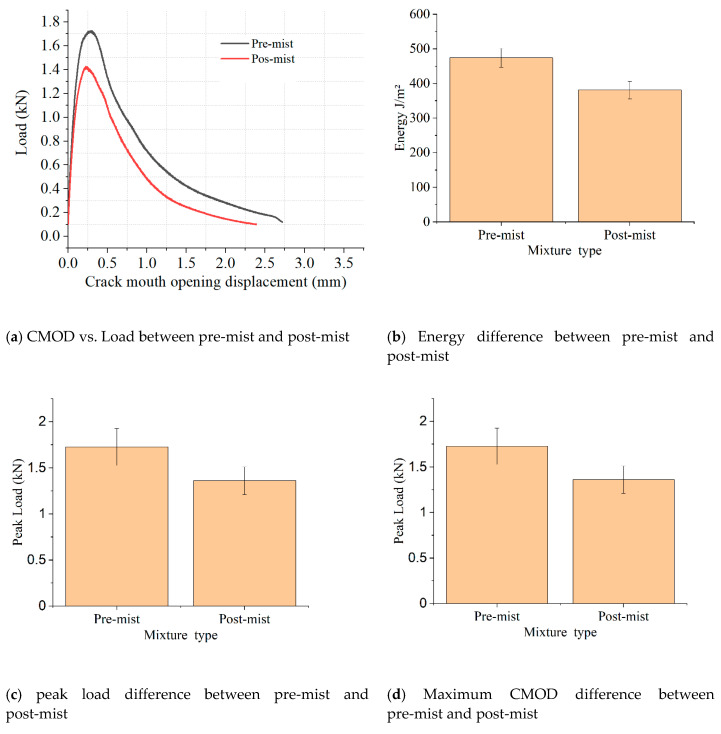
Low-temperature cracking resistance results of pre-mist and post-mist CIR asphalt mixture: (**a**) CMOD vs. Load between pre-mist and post-mist; (**b**) Energy difference between pre-mist and post-mist; (**c**) peak-load difference between pre-mist and post-mist; (**d**) Maximum CMOD difference between pre-mist and post-mist.

Low-temperature cracking resistance was evaluated by the DCT test, which investigated the implications of the CIR mixture on low-temperature cracking. The fracture energy directly expressed the cracking performance of the asphalt mixture, and the mixture with the higher fracture energy had a better low-temperature cracking property. The fracture energy of the pre-mist and post-mist CIR mixture is displayed in Figure 12b. Pre-mist CIR mixture showed better low temperature cracking resistance compared with the post-mist CIR mixture. The recommended low-temperature crack resistance threshold determined by MnDOT is 400 J/m^2^ [33]. The peak load of the DCT test defines the force needed to initiate the cracking in the asphalt mixture at low temperatures. The peak load represents the cracking propagation possibility of the asphalt mixture; an asphalt mixture with a higher peak load was harder to crack. The peak load of the two asphalt mixtures under different temperatures is shown in Figure 12c. The maximum CMOD represents the deformation that occurs in the asphalt mixture during the DCT test. The asphalt mixture with a higher maximum CMOD had better deformation ability under low temperatures. The maximum CMOD of the pre-mist and post-mist CIR mixture is displayed in Figure 12d. The pre-mist CIR mixture showed better low temperature cracking resistance compared with the post-mist CIR mixture. The results of the DCT test were consistent with the results obtained from creep compliance. 

### 3.4. DSR results

**Figure 13:**
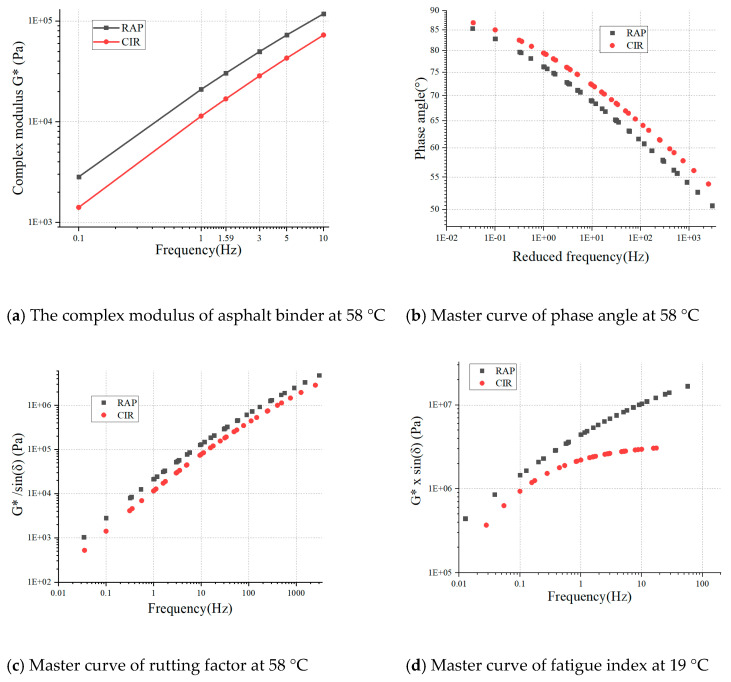
DSR test results for RAP and CIR asphalt: (**a**) The complex modulus of asphalt binder at 58 °C; (**b**) master curve of phase angle at 58 °C; (**c**) master curve of rutting factor at 58 °C; (**d**) master curve of fatigue index at 19 °C.

The complex modulus of RAP asphalt and CIR asphalt under six frequencies at 58 °C were displayed in Figure 13a. RAP and CIR asphalt were extracted from the loose materials. Asphalt extracted from the loose materials did not need any aging to simulate the short-term and long-term performance. It was determined that the presence of emulsion asphalt decreased the complex modulus because it made the asphalt softer compared with RAP. Simultaneously, the complex modulus of CIR and RAP asphalt increased with as the frequency increased. For example, the complex modulus of the RAP asphalt binder was 20.95 kPa at 1.00 Hz, and it increased 44.97% and 464.21% to 1.59 Hz and 10.00 Hz, respectively. The reason for this is that asphalt shows higher elastic components and properties with the increased frequency. In contrast, a lower frequency, which represents a longer loading time, generally causes the asphalt to display more viscous properties. This evidence could be used to reveal the reason that rutting distress appears more easily on longitudinal-slope asphalt pavement over long distance. An automobile would generally be driven at low speed over a long time, thus producing low-frequency-related properties. 

Figure 13b illustrates the master curves of phase angles at 58 °C. As the phase angle decreased, the viscous component of asphalt decreased, and the elastic component increased. The addition of emulsified asphalt increased the phase angle of the asphalt. The master curves of the rutting factor for RAP asphalt and CIR asphalt at 58 °C are shown in Figure 13c. It is worth mentioning that low temperature corresponded to the highly reduced frequency. It was revealed that the rutting index increased with increasingly reduced frequency. Meanwhile, the asphalt had more elastic components and a higher resistance to deformation under low-temperature conditions. This was consistent with the fact that a lower temperature indicated a higher rutting coefficient and better resistance to deformation. For example, adding emulsion to the RAP asphalt binder caused the rutting factor value to decrease throughout the reduced frequency, which indicated that the stiffness of the asphalt binder had been reduced compared to that of the RAP asphalt. The master curves of fatigue factor for the two types of asphalt at the reference temperature of 19 °C are presented in Figure 13d. In order to resist fatigue cracking, an asphalt binder should be elastic (able to dissipate energy by rebounding, not cracking) but not too stiff (excessively stiff substances will crack rather than deform and rebound). Therefore, the complex shear modulus viscous portion |G*|×sinδ should be minimal. It is clear that the addition of emulsified asphalt in the RAP asphalt decreased the |G*|×sinδ value of the asphalt, which may improve the resistance of fatigue cracking properties.

### 3.5. ABCD Results

**Figure 14:**
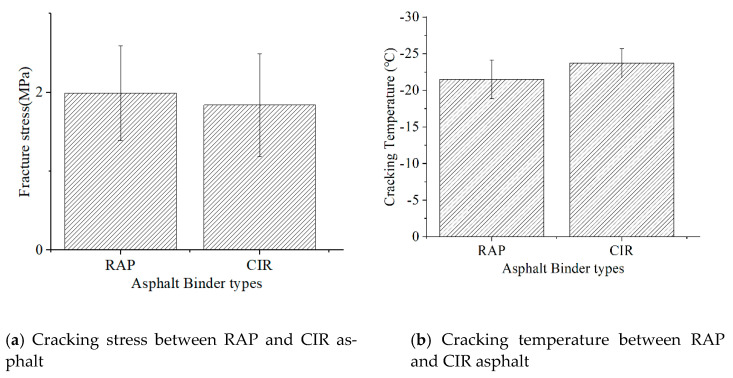
Asphalt cracking stress and temperature between RAP and CIR: (**a**) Cracking stress between RAP and CIR asphalt; (**b**) Cracking temperature between RAP and CIR asphalt.

In this section, the effect of added emulsion on the low-temperature performance of the asphalt binder was evaluated. According to AASHTO TP 92-11, the cracking temperature was determined based on the strain jump when the fracture stress was recorded. The test results are shown in Figure 14. It should be noted that the addition of emulsion improved the low-temperature cracking resistance of asphalt. It not only increased the fracture stress but also decreased the cracking temperature. For example, the cracking temperature of CIR with the added emulsion decreased from −26.45 °C to −29.2 °C. The main reason was that the asphalt emulsion made the CIR asphalt binder softer than the RAP asphalt binder. Since CIR asphalt is softer than RAP asphalt, it showed a lower cracking temperature, while RAP asphalt had higher fracture stress.

### 3.6. Pavement Distress Prediction by Pavement M-E Design

A pavement M-E design was conducted to predict pavement distress [35,36]. The climate data of the place near Dickinson County was used in this study according to the M-E used-guide recommendation. The dynamic modulus from the asphalt mixture and complex shear modulus from the asphalt binder was the input material property that could be used for distress predictions. As described in the method section, the pavement M-E design was used as the pavement distress predictor in this paper because it uses dynamic modulus values and complex shear modulus values to obtain the master curve for asphalt concrete layers. The dynamic modulus values of pre-mist and post-mist CIR asphalt mixture were measured, and the complex shear modulus (extracted from the RAP and CIR) was measured by DSR equipment used in the M-E pavement analysis. The pavement distress prediction results were analyzed based on the dynamic modulus of CIR (pre-mist and post-mist). The complex shear modulus of CIR asphalt was used to do the M-E analysis.

The pavement distress prediction results of the pre-mist and post-mist CIR mixture dynamic modulus are shown in Figure 14. It should be mentioned that the dynamic modulus of pre-mist and post-mist CIR mixture reflected the effect of moisture damage in the CIR pavement layer on the pavement distress predictions, The M-E input parameters of other pavement layers (top, base, and subbase) were based on the reference value from the report [37]. In reality, the moisture damage not only caused deterioration to the leveling layer, but it also affected the top, base, subbase, and subgrade layers. The distress prediction between pre-mist and post-mist CIR mixture in this study only revealed the maximum moisture damage of the CIR layer. Rut depths of the post-mist CIR pavement layer were 0.34 inches higher compared with the pre-mist mixture, which was caused by a reduction in stiffness. It shows an analogous trend with the rutting-factor results based on asphalt mixture dynamic modulus results. Bottom-up fatigue cracking results of the post-mist CIR pavement layer increased by 6% compared with the pre-mist CIR pavement layer, which implied a similar tendency with the creep compliance and disk-shaped compact tension test results. Furthermore, the international roughness index of the post-mist CIR pavement layer increased by 10% compared with the pre-mist CIR pavement layer. The results of the average dynamic modulus at pre-mist and post-mist conditions are shown in Table 2. Overall, the moisture damage of the CIR asphalt mixture deteriorated high- and low- temperature performance but it was acceptable when used in a low-volume road.

**Figure 15:**
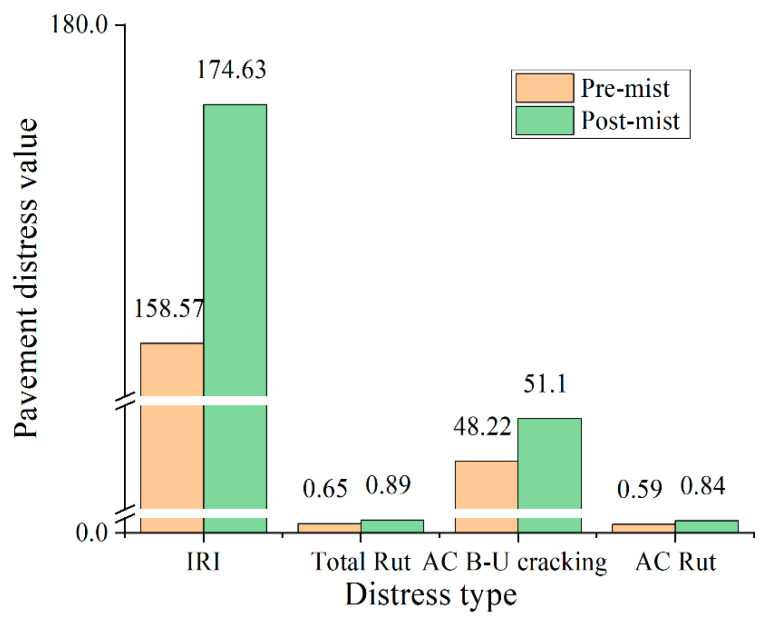
Pavement distress prediction by the dynamic modulus of CIR (pre-mist and post- mist). Note: IRI smoothness (in/mile or 0.016 m/km), Total Rut (in or 0.0254 m), AC B-U(bottom-up) cracking (% lane area), AC Rut (in or 0.0254 m), only consider the difference of 12.7 cm CIR layers.

The pavement distress prediction results of RAP and CIR complex shear modulus are shown in Figure 15. It was found that CIR asphalt increased the rut depths of the total pavement and asphalt layer, which was caused by a reduction in stiffness. However, bottom-up fatigue cracking results decreased by 1.34%. This showed a similar trend compared with the rutting and fatigue factors from the DSR results. Moreover, the international roughness index (IRI) of CIR asphalt decreased when compared with the RAP asphalt. It should be mentioned that the results just considered the DSR data (RAP and CIR asphalt) on 12.7 cm CIR layers difference. The input value of other layers was based on the reference value from the report [37,38]. The DSR results of RAP and CIR used in this study are shown in Table 3. The pavement distress prediction results were studied by the complex shear modulus (RAP and CIR). The dynamic modulus of the pre-mist CIR asphalt mixture was used to do the M-E analysis. In fact, the dynamic modulus of the RAP asphalt mixture should be used for the M-E design, so the M-E results in Figure 15 did not completely reflect the predicted distress.

**Figure 16:**
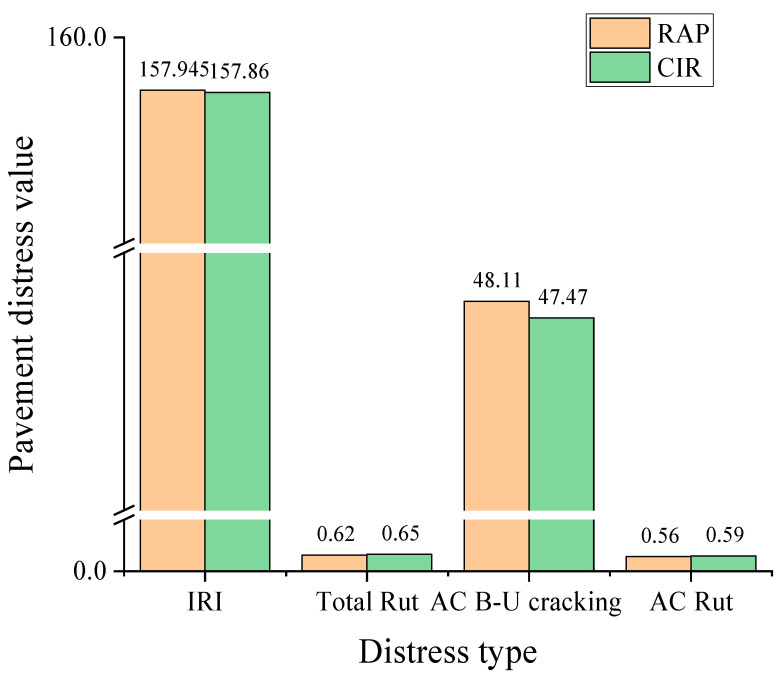
Pavement predicted distresses to complex shear modulus (RAP and CIR). Note: Note: IRI smoothness (in/mile or 0.016 m/km), Total Rut (in or 0.0254 m), AC B-U(bottom-up) cracking (% lane area), AC Rut (in or 0.0254 m), only consider the difference of 12.7 cm CIR layers.

The pavement distress prediction results of the RAP and CIR complex shear modulus are shown in Figure 16. It could be founded that CIR asphalt increased the rut depths of the total pavement and asphalt layer, which was caused by a reduction in stiffness. However, bottom-up fatigue cracking results decreased by 1.34%. It showed a similar trend compared with the rutting and fatigue factors from the DSR results. Moreover, the international roughness index (IRI) of CIR asphalt decreased when compared with the RAP asphalt. It should be mentioned that the results just considered the DSR data (RAP and CIR asphalt) on 12.7 cm CIR layers difference. The input value of other layers was based on the reference value from the report [36]. The pavement distress prediction results were studied by complex shear modulus (RAP and CIR). The dynamic modulus of the pre-mist CIR asphalt mixture was used to do the M-E analysis. In fact, the dynamic modulus of the RAP asphalt mixture should be used for the M-E design, so the M-E results in Figure 16 could not completely reflect the prediction distress.

## 4. Field Construction and Pavement Condition Assessment

**Figure 17:**
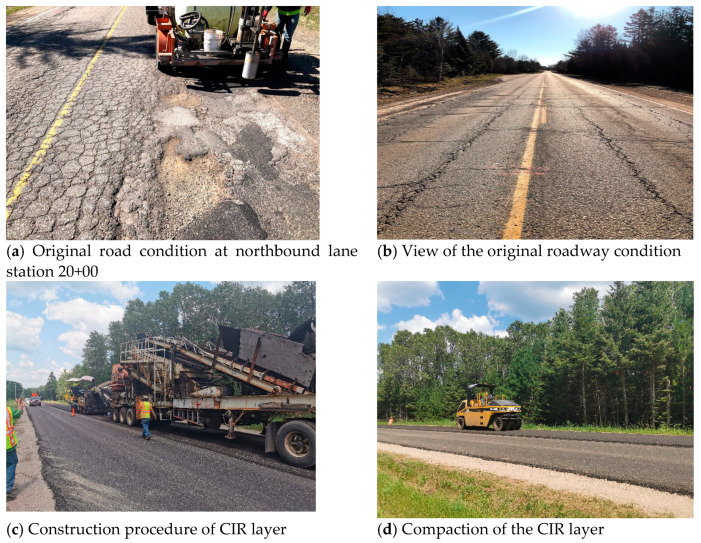
Original roadway condition and construction procedure: (**a**) Original road condition at northbound lane station 20+00; (**b**) View of the original existing roadway condition; (**c**) Construction procedure of CIR layer; (**d**) Compaction of the CIR layer.

A demonstration project was paved by The Dickinson County Road Commission in Dickinson, Michigan, in July 2020. The original roadway condition is shown in Figure 17a,b. It was poor, and the surface layer was milled before the new pavement was placed. The construction procedures in the field are shown in Figure 17c,d. The use of cold in-place recycling restored the old pavement to the desired profile, eliminated existing wheel ruts, restored the crown and cross slope, and eliminate potholes, irregularities, and rough areas. It also eliminated transverse, reflective, and longitudinal cracks. The asphalt emulsion was applied with the milling loose asphalt mixture, and it was placed in the original place as the level layer. After the CIR asphalt pavement layer was compacted, a new top layer was paved over it. The service condition of the road will be estimated in the future.

## 5. Summary and Conclusions

This study concentrated on characterizing the CIR asphalt mixture moisture susceptibility performance and extraction of asphalt binder properties to predict the pavement distress and deterioration based on a mechanistic–empirical pavement design. Asphalt mixture moisture susceptibility performance (pre-mist and post-mist) was estimated by the dynamic modulus test, DCT test, and asphalt-binder performance (RAP and CIR asphalt obtained from the standard solvent extraction process). Higher-temperature and medium-temperature performances were evaluated by DSR equipment and low-temperature properties were estimated by the ABCD. Then the M-E pavement design was conducted to predict the pavement distress. The following conclusions are summarized from this study:

(1) The dynamic modulus test showed that the moisture condition has a significant effect on the CIR asphalt mixture, especially with high temperature and low frequency. The dynamic modulus of the post-mist asphalt mixture decreased 13–43% at various temperatures and frequencies, but pavement M-E results showed that moisture damage of the CIR layer only increased AC Rut and total Rut by 0.34 inches at low traffic volume. It showed that CIR asphalt pavement may still be used for low-volume roads.

(2) Compliance creep, and disk-shaped compact tension test results showed that MIST conditioning decreased the low temperature cracking resistance of the asphalt mixture, and the low-temperature cracking energy of the CIR asphalt mixture decreased 20% between pre-mist and post-mist condition. The pavement M-E results showed that moisture damage to the CIR layer only increased bottom-up cracking by 2.9%. The lab test results suggest that the performance of CIR materials may have poor performance, but the M-E results showed that it is possible to apply CIR materials on low traffic-volume roads.

(3) The asphalt binder that included emulsion asphalt made the asphalt softer and increased fatigue property and low temperature cracking resistance while decreasing rutting resistance performance.

(4) Pavement M-E analysis based on DSR results of RAP and CIR binder showed that the asphalt binder with emulsion increased the rutting predictions while reducing the IRI, bottom-up fatigue-cracking predictions.

In summary, the implementation of CIR technology in the project improved the low-temperature cracking and fatigue performance in asphalt pavement. Meanwhile, the moisture damage to the CIR asphalt mixture accelerated the high-temperature rutting and low-temperature cracking performance, but it is possibly acceptable when used for low-volume roads.

## Figures and Tables

**Table 1:** Mix design for CIR asphalt mixture.

Test Parameters	Test Results			Specification Requirement
Emulsion Content	2.0%	2.5%	3.0%	-
Optimum Water for Mixing	2.0%	2.0%	2.0%	-
Bulk Specific Gravity	2.110	2.125	2.132	-
Maximum Theoretical Specific Gravity	2.443	2.440	2.436	-
Air Voids	13.6%	12.9%	12.5%	-
Marshall Stability (kN)	7.80	7.16	7.28	5.56 kN min
Conditioned Marshal Stability (kN)	5.74	5.47	5.12	-
Retained Stability	74%	76%	70%	70% min
Raveling Test	1.61%	0.30%	0.19%	2% Max

**Table 2:** Average dynamic modulus value pre-mist and post-mist condition.

|E*| (MPa) Average Value Pre-Mist Condition							
	F (Hz)	0.1	0.5	1	5	10	25
**T (°C)**							
−10		7098.3	8737.7	9418.5	10,529.2	11,181.8	12,379.7
10		2270.4	3125.8	3572.8	4873.0	5404.6	6018.4
21		623.6	956.2	1216.3	2037.7	2505.0	2979.3
37		320.2	475.5	664.4	1047.0	1341.7	1567.5
	|E*| (MPa) Average Value Post-Mist Condition						
	**F (Hz)**	**0.1**	**0.5**	**1**	**5**	**10**	**25**
**T (°C)**							
−10		5363.1	7020.6	7789.8	9318.7	9902.8	10,851.1
10		1773.2	2591.1	2851.7	3864.8	4347.0	4898.0
21		440.6	710.4	1015.3	1556.9	1821.7	2189.7
37		198.0	296.8	429.8	669.4	764.8	999.5

**Table 3:** DSR results of RAP and CIR used in this study.

Frequency @ 10 rad/s for RAP		
T (°C)	|G*| (Pa)	Phase Angle°
13	30,628,000	35
25	70,84,800	46
34	3,888,633	53.6
46	621,153	62.67
58	114,973	70.4
**Frequency @ 10 rad/s for CIR**		
T (°C)	|G*| (Pa)	Phase Angle°
13	14,437,000	36
25	56,78,100	45
34	2,281,967	57.3
46	354,476	66.7
58	68,359	73.3

## Data Availability

The datasets generated during analyzed during the current study are available from the corresponding author on reasonable request.
